# Learning More About the Effects of Gamification on Physical Activity. Comment on “Evaluating the Effectiveness of Gamification on Physical Activity: Systematic Review and Meta-analysis of Randomized Controlled Trials”

**DOI:** 10.2196/36396

**Published:** 2022-05-03

**Authors:** Cheng-Hsien Hung, Yung-Shuo Kao

**Affiliations:** 1 Department of Pharmacy Chang Bing Show Chwan Memorial Hospital Changhua County Taiwan; 2 Department of Radiation Oncology China Medical University Hospital Taichung Taiwan

**Keywords:** behavior change, eHealth, gamification, health behavior, intervention, meta-analysis, mobile phone, physical activity, systematic review, elderly, old adults

We read with great interest the article, “Evaluating the Effectiveness of Gamification on Physical Activity: Systematic Review and Meta-analysis of Randomized Controlled Trials” by Mazeas et al [1]. The authors conducted a systematic review and meta-analysis to evaluate the effectiveness of gamification on physical activity (PA). This meta-analysis confirms that gamified interventions are promising for promoting PA in various populations. Although the authors acknowledge the limitations of their study, we wish to highlight several methodological issues and provide our perspective.

First, Paul et al [2] was not a randomized controlled trial, but a nonrandomized clinical controlled trial. As mentioned in both the methods and limitations of this study, the authors used nonrandomized allocation. According to the Cochrane Handbook for Systematic Reviews of Interventions [3], “Predefined, unambiguous eligibility criteria are a fundamental prerequisite for a systematic review.” Authors should select literature strictly according to inclusion and exclusion criteria, especially for the quantitative analysis. Mixing research with different study designs may significantly affect the results and the level of evidence.

Second, the total number of hours of gamification performed can make a significant difference. Although the authors have conducted a subgroup analysis of the duration of gamification (short- and long-term interventions), each type of gamification is different, and we do not know the number of minutes of gamification performed per day. The total number of hours of gamification performed can vary greatly across the different studies. We believe this could be a potential source of heterogeneity.

Third, this meta-analysis may not apply to older adults. The mean age of participants in the selected studies was 35.7 years, and most of the studies were conducted on participants aged <65 years. The American College of Sports Medicine suggests that the population most in need of exercise may be older adults [4]. In a previous meta-analysis, the lack of PA was associated with all-cause mortality and cardiovascular mortality, fractures, and falls among older populations [5]. However, this age group may have difficulty with device operation and gamification rules. Age may affect the gamification experience, leading to limitations in the application of evidence.

In conclusion, we believe that clarification of the above points can strengthen the interpretation of the study results. The authors have analyzed an important issue. A better understanding of the effects of gamification mechanisms on PA is critical for clinicians.

## Abbreviations

PA: physical activity
